# Prognostic Role of Albumin, Bilirubin, and ALBI Scores: Analysis of 1000 Patients with Hepatocellular Carcinoma Undergoing Radioembolization

**DOI:** 10.3390/cancers11060879

**Published:** 2019-06-24

**Authors:** Mark Antkowiak, Ahmed Gabr, Arighno Das, Rehan Ali, Laura Kulik, Daniel Ganger, Christopher Moore, Michael Abecassis, Nitin Katariya, Samdeep Mouli, Devalingam Mahalingam, Robert J. Lewandowski, Riad Salem, Ahsun Riaz

**Affiliations:** 1 Section of Interventional Radiology, Department of Radiology, Northwestern University, Chicago, IL 60611, USA; mark.antkowiak@northwestern.edu (M.A.); ahmed.gabr@northwestern.edu (A.G.); arighno.das@northwestern.edu (A.D.); rehanali253@gmail.com (R.A.); s-mouli@northwestern.edu (S.M.); r-lewandowski@northwestern.edu (R.L.); r-salem@northwestern.edu (R.S.); 2 Division of Hepatology, Department of Medicine, Northwestern University, Chicago, IL 60611, USA; lkulik@nm.org (L.K.); d-ganger@northwestern.edu (D.G.); christopher.moore@northwestern.edu (C.M.);; 3 Division of Transplant Surgery, Department of Surgery, Northwestern University, Chicago, IL 60611, USA; mabecass@northwestern.edu (M.A.); nitin.katariya@northwestern.edu (N.K.); 4 Division of Oncology, Department of Medicine, Northwestern University, Chicago, IL 60611, USA; mahalingam@northwestern.edu

**Keywords:** albumin, bilirubin, radioembolization, prognosis

## Abstract

**Introduction:** We compared the efficacy of the ALBI (albumin–bilirubin) score to the established Child–Pugh (CP) grade in hepatocellular carcinoma (HCC) patients treated with yttrium-90 radioembolization (Y90). We further assessed the individual contributions of albumin and bilirubin to survival prediction. **Methods:** 1000 consecutive HCC patients treated with Y90 were included. Overall survival (OS) was assessed using Kaplan Meier analysis. Sub-stratification analyses were performed using CP and ALBI and in subgroups determined by United Network for Organ Sharing (UNOS) or Barcelona Clinic Liver Cancer (BCLC) staging. The independent impact (hazard ratio (HR)) of ALBI, CP, albumin, and bilirubin on survival was assessed using Cox proportional hazards analysis. **Results:** Median OS for ALBI 1, 2, and 3 grades was 46.7, 19.1, and 8.8 months, respectively. The HR for death for ALBI 2 vs. ALBI 1 was 3.39 (1.75–6.57); ALBI 3 vs. ALBI 1 was 7.58 (3.89–14.79); and the c-index was 0.623. Median OS for CP A, B, and C was 21.7, 11.3, and 6.0 months, respectively. The HR for death for CP B vs. CP A was 2.04 (1.71–2.43); CP C vs. CP A was 3.27 (2.08–5.14); and the c-index was 0.616. Stratified OS showed unique prognostic groups identified by ALBI within CP-B and CP-C. Median OS for albumin grades 1, 2, and 3 was 46.0, 17.1, and 9.1 months, respectively. Median OS for bilirubin grades 1, 2, and 3 was 15.6, 21.0, and 5.8 months, respectively. The HR for death for albumin 2 vs. 1 was 2.48 (1.81–3.41); albumin 3 vs. 1 was 4.74 (3.44–6.54); and the c-index was 0.640. The HR for death for bilirubin 2 vs. 1 was 1.09 (0.82–1.44); bilirubin 3 vs. 1 was 2.37 (1.66–3.40); and the c-index was 0.533. **Conclusions:** ALBI outperforms CP in survival prognosis in Y90 treated patients. On sub-analyses, serum albumin (not bilirubin) appears to be the main driver of survival prediction. Our study supports the prognostic ability of ALBI and may suggest a role of albumin alone as a biomarker for patients with HCC.

## 1. Introduction

Hepatocellular carcinoma (HCC) is a common cancer worldwide and is associated with significant morbidity and mortality [1]. HCC often occurs in the setting of cirrhosis, which can complicate treatment and is associated with poor prognosis. In providing patient care, treatment must be both tolerated and beneficial. Expected patient response to treatment can be assessed using prognostic clinical tools that assess both tumor burden and liver function.

Currently, patients with HCC are staged using the Barcelona Liver Center (BCLC) staging system. BCLC relies on Child–Pugh (CP) class to assess liver function [2]. Application of CP to HCC patients has important limitations, including subjective measures of liver function (ascites, encephalopathy), duplicative measures (albumin, ascites), and information loss through conversion of continuous metrics to discrete metrics (albumin, bilirubin, international normalized ration (INR)). It is becoming increasingly clear that that CP grade is not optimal for assessing liver failure in patients with HCC and a superior measure may lead to better patient outcomes [2,3]. 

Johnson et al., proposed an alternative to CP based on serum albumin and bilirubin levels alone (ALBI). ALBI utilizes albumin and bilirubin as continuous metrics and weighs each according to a validated statistical model. Albumin and bilirubin are objective and hence widely used liver function tests. ALBI is emerging as a potentially powerful new tool for the assessment of liver function in patients with HCC in place of CP [4]. In this study, we examine the role of ALBI in 1000 patients with HCC treated with yttrium-90 radioembolization (Y90). We further assess the independent prognostic contributions of albumin and bilirubin to the ALBI score to determine impact on overall survival.

## 2. Results

### 2.1. Overall Characteristics

A total of 1000 patients with a median age of 65 were included in the study. Baseline characteristics are presented in Table 1.

### 2.2. ALBI Compared with CP

#### 2.2.1. Counts

ALBI and CP grades were calculated for all 1000 patients. 71 patients were classified as ALBI 1 (7.1%), 637 as ALBI 2 (63.7%), and 292 as ALBI 3 (29.2%); 506 patients were classified as CP A (50.6%), 450 as CP B (45.0%), and 44 as CP C (4.4%).

#### 2.2.2. Survival

Survival analysis was performed for ALBI and CP grades. Median survival for ALBI 1, 2, and 3 patients was 46.7 (95% confidence interval (CI): 43.8–not reached), 19.1 (16.1–21.5), and 8.8 (6.9–10.6) months, respectively; Median survival of CP A, B, and C patients was 21.7 (CI: 19.2–27.9), 11.3 (10.1–13.3), and 6.0 (4.6–not reached) months, respectively.

Stratified survival analysis was performed for ALBI within CP grades and CP within ALBI grades. Results are presented in Table 2 (survival curves shown in Supplemental Figure S1). Log rank analysis yielded a X^2^ of 12.5 (*p*-value = 0.001) for ALBI within CP A, and a X^2^ of 20.2 (*p*-value < 0.001) for ALBI within CP B; log rank analysis yielded a X^2^ of 7.7 (*p*-value = 0.006) for CP within ALBI 2 and a X^2^ of 1.0 (*p*-value = 0.314) for CP within ALBI 3. Log rank analysis was not performed for ALBI within CP C or for CP within ALBI 1 due to low patient count.

#### 2.2.3. UNOS Counts

The United Network for Organ Sharing (UNOS) stage was calculated for all 1000 patients. Preliminary analysis showed stratification by T1 and T2 stages or stratification by T4b, N, and M stages resulted in statistically similar curves (Supplemental Figure S3). These groups were hence combined into T1/T2 and T4b/N/M. Three hundred and sixty-eight patients were classified as T1/T2 (36.8%), 169 as T3 (16.9%), 147 as T4a (14.7%), and 316 as T4b/N/M (31.6%). ALBI and CP counts within UNOS stages are shown in Table 3.

#### 2.2.4. UNOS Survival

Survival analysis was performed for UNOS stages. Median survival of UNOS T1/T2, T3, T4a, and T4b/N/M patients was 33.5 (29.1–47.3), 29.6 (22.1–38.3), 13.2 (10.5–16), and 7.4 (6.5–8.4) months, respectively.

Stratified survival analysis was performed for ALBI or CP within UNOS grades. Results are presented in Table 3 (survival curves shown in Supplemental Figure S2). Differences between ALBI and CP were most pronounced in T3. In T3 patients, median survival for ALBI 1, 2, and 3 was 43.8 (14.0–not reached), 35.0 (24.9–41.6), and 11.2 (6.8–not reached) months, respectively; Median survival for CP A, B, and C patients was 35.7 (25.5–44.4), 19.9 (14.7–38.3), and 14.8 (not reached–not reached) months respectively.

#### 2.2.5. Cox Proportional Hazards

Univariate Cox proportional hazards analysis was performed to assess the impact of ALBI and CP grade on overall survival. Results are presented in Table 4.

The hazard ratio (HR) for death of ALBI 2 compared to ALBI 1 was 3.39 (1.75–6.57); ALBI 3 compared to ALBI 1 was 7.58 (3.89–14.79); CP B compared to CP A was 2.04 (1.71–2.43); and CP C compared to CP A was 3.27 (2.08–5.14). The Cox model built on ALBI had a c-index of 0.623 and an Akaike information criterion (AICc) of 6155.8 and the model built on CP had a c-index of 0.616 and an AICc of 6184.7.

### 2.3. Albumin Compared to Bilirubin

#### 2.3.1. Counts

Albumin and bilirubin levels were assessed for all 1000 patients. One hundred and eighty-eight patients were classified as albumin 1 (18.8%), 490 as albumin 2 (49.0%), and 322 as albumin 3 (32.2%); 824 patients were classified as bilirubin 1 (82.4%), 124 as bilirubin 2 (12.4%), and 52 as bilirubin 3 (5.2%).

#### 2.3.2. Survival

**Figure 1:**
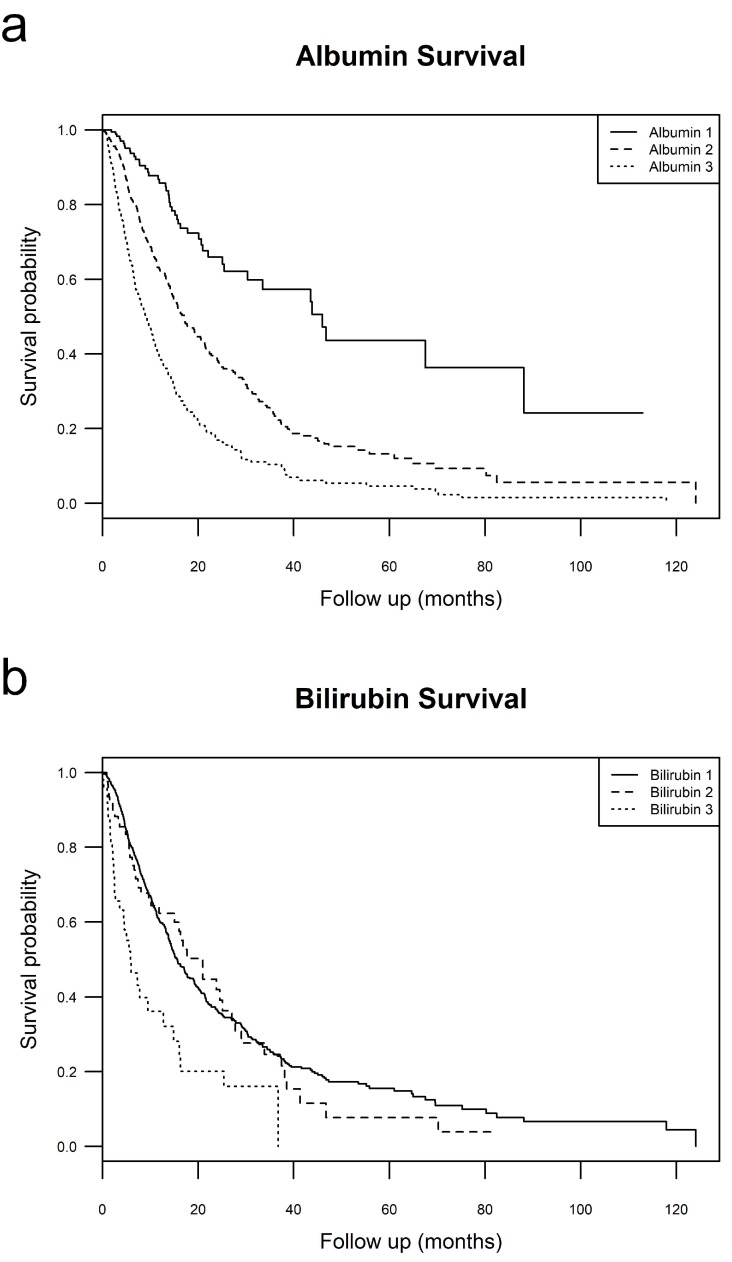
Albumin and bilirubin Survival. (**a**-**top**) Kaplan Meier survival curves of all patients stratified by albumin grade; (**b**-**bottom**) Kaplan Meier survival curves of all patients stratified by bilirubin grade.

Survival analysis was performed for albumin and bilirubin groups. Median survival for albumin 1, 2, and 3 patients was 46.0 (30.3–not reached), 17.1 (14.9–20.6), and 9.1 (7.6–10.8) months, respectively. Median survival of bilirubin 1, 2, and 3 was 15.6 (14.4–17.7), 21.0 (15.1–27.8), and 5.8 (4.4–14.8) months, respectively. This is shown in Figure 1.

#### 2.3.3. BCLC Counts

BCLC stage was calculated for all 1000 patients. Two hundred and sixty-three patients were classified as A (26.3%), 152 as B (15.2%), 541 as C (54.1%), and 44 as D (4.4%). Albumin and bilirubin counts within BCLC stages are shown in Table 5.

#### 2.3.4. BCLC Survival

Survival analysis was performed on BCLC stages. Median survival of BCLC A, B, C, and D patients was 35.1 (30.2–45.0), 19.0 (15.4–25.1), 11.3 (9.7–13.1), and 6.0 (4.6–not reached) months, respectively.

**Figure 2:**
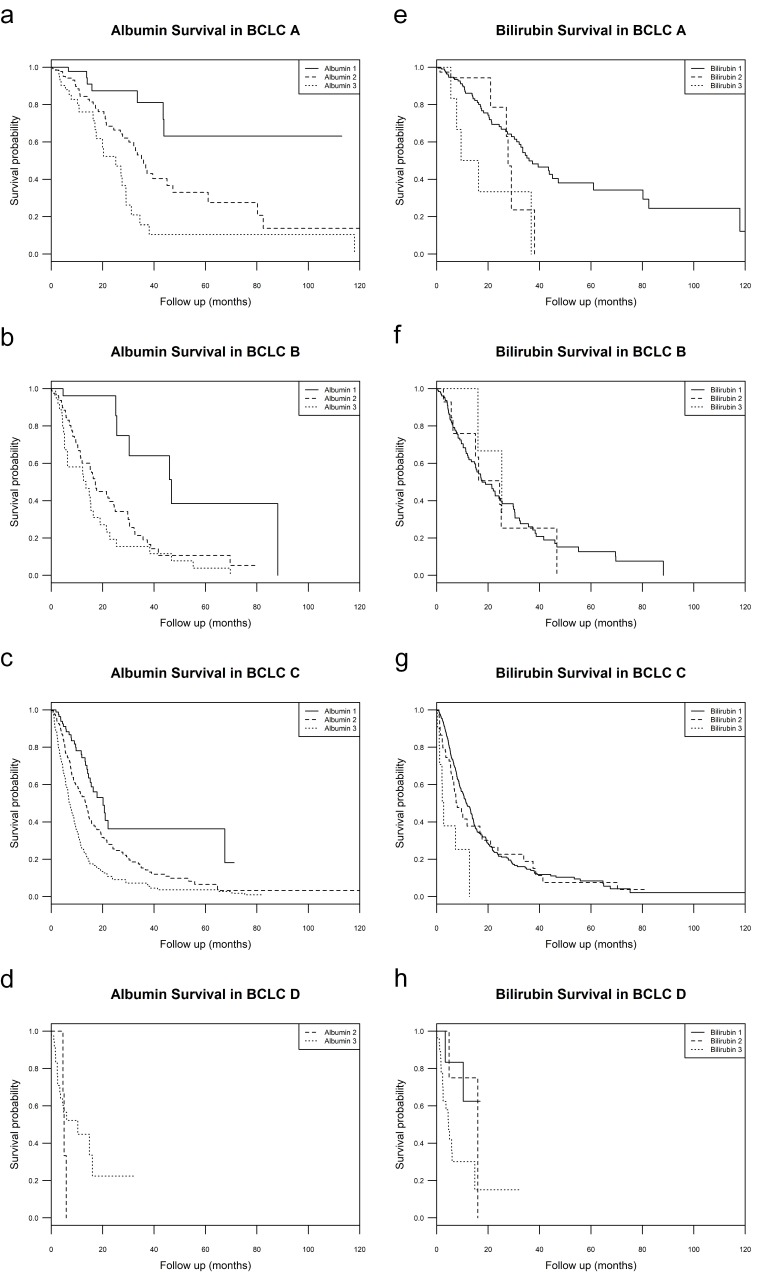
Albumin and bilirubin survival within BCLC stages. (**a**–**d**, **top left**–**bottom left**) Kaplan Meier survival curves of patients in each BCLC stage stratified by albumin grade; (**e**–**h**, **top right**–**bottom right**) Kaplan Meier survival curves of patients in each BCLC stage stratified by bilirubin grade.

Stratified survival analysis was performed for albumin or bilirubin groups within BCLC grades. Results are presented in Table 5 (survival curves are shown in Figure 2). Differences were most notable in BCLC B patients. In BCLC B patients, median survival for albumin 1, 2, and 3 was 46.7 (30.3–not reached), 17.2 (12.0–24.9), and 13.5 (6.3–21.4) months, respectively; median survival for bilirubin 1, 2, and 3 was 17.7 (15.1–25.5), 24.5 (15.1–not reached), and 25.4 (16.1–not reached) months, respectively. Log rank analysis yielded a X^2^ of 21.2 (*p*-value < 0.001) for albumin within BCLC B, and a X^2^ of 0.1 (*p* value = 0.90) for bilirubin within BCLC B. Stratified survival analysis was also compared between albumin or ALBI within BCLC grades.

#### 2.3.5. Cox Proportional Hazards Analysis

Univariate Cox proportional hazards analysis was performed to assess the impact of albumin and bilirubin groups on overall survival. Results are presented in Table 4. The HR for death for albumin 2 compared to 1 was 2.48 (1.81–3.41); albumin 3 compared to albumin 1 was 4.74 (3.44–6.54); and the c-index was 0.640 and the AICc was 6136.6. The HR for death of bilirubin 2 compared to bilirubin 1 was 1.09 (0.82–1.44); bilirubin 3 compared to 1 was 2.37 (1.66–3.40); and the c-index was 0.533 and the AICc was 6240.9.

Multivariate Cox proportional hazards analysis was performed to compare the impact of albumin or ALBI with BCLC. With BCLC, the HR for death for albumin 2 compared to albumin 1 was 2.31 (1.69–3.17); and albumin 3 compared to albumin 1 was 4.03 (2.91–5.58). The HR for death of ALBI 2 compared to ALBI 1 was 2.89 (1.58–2.95); and ALBI 3 compared to 1 was 5.93 (3.03–11.61). The Cox model built on albumin with BCLC had a c-index of 0.703 and an AICc of 6045.6, and the model built on ALBI with BCLC had a c-index of 0.697 and an AICc of 6061.1.

These differences can also be seen with albumin and bilirubin as continuous variables (Table 6). In the entire cohort, the hazard ratio for albumin was 0.34 (0.29–0.40) and bilirubin was 1.00 (0.98, 1.02). For UNOS T1/T2, T3, T4a, T4b/N/M subgroups respectively, the hazard ratios for albumin were 0.26, 0.21, 0.46, and 0.47 and for bilirubin were 1.27, 0.98, 1.42, and 1.61. Notably, in UNOS T3, the hazard ratio for bilirubin was not significant.

## 3. Discussion

ALBI is becoming increasingly recognized as a valuable tool in evaluating patients with HCC [4,5,6,7,8,9,10]. ALBI was originally designed by Johnson et al. to assess liver function in patients with liver cancer and cirrhosis. It was built on a cohort of 1313 Japanese patients with HCC or cirrhosis and validated on cohorts from Japan, China, the United Kingdom, and the United States [11]. As opposed to CP grade, ALBI relies solely on serum albumin and bilirubin, which are readily collected in standard blood tests. ALBI uses albumin and bilirubin as continuous measures and weighs the contribution of each factor accordingly. In contrast, CP staging simplifies continuous measures into ordinal values [2,3]. This results in information loss because the contribution and magnitude of each factor (albumin, bilirubin, INR, ascites, and encephalopathy) are simplified into ordinal values between 1 and 3. Furthermore, some of these factors are correlated and thus duplicative (albumin, ascites) and some are subjective (ascites, encephalopathy). Thus, in contrast to ALBI, CP suffers from information loss, duplication, and subjectivity.

In this study, we show that: (a) ALBI is effective in prognosticating survival and (b) albumin alone can independently be used to prognosticate survival.

ALBI divided patients into 3 groups with statistically different median survivals (ALBI 1: 46.7, ALBI 2:19.2, ALBI 3:8.8 months), with a log rank X^2^ of 108 (*p*-value < 0.001). In contrast, median survival of CP B and CP C patients was not statistically different (CP B: 11.3, CP C: 6.0 months), with a log rank test X^2^ of 3.3 (*p*-value = 0.069). Importantly, ALBI identified a unique group of patients (ALBI 1) who had longer predicted median survival, a group not identified by CP. Patients identified as ALBI 1 may represent a sub-select group of patients with very good survival (median survival 46.7 months).

We next examined the prognostic value of ALBI within each CP grade. Notably, ALBI split CP A patients into groups with statistically different median survival with a log rank X^2^ of 12.5 (*p*-value < 0.001) and identified a cohort of patients within CP A with markedly better survival at 46.7 months as compared to 21.7 months overall for CP A patients. In contrast, CP appeared to hold less prognostic value within ALBI grades; in ALBI 3 patients stratified by CP, median survival in CP B and CP C patients was not statistically different with a log rank X^2^ of 1.0 (*p*-value = 0.314). Both ALBI and CP identified groups with statistically different median survivals within UNOS.

Cox proportional hazards analysis further demonstrated the prognostic power of ALBI. Univariate analysis with ALBI and CP showed significantly greater hazard ratios in ALBI (ALBI 2:1 was 3.39, ALBI 3:1 was 7.58) than CP (CP B:A was 2.04, CP C:A was 3.27), indicating ALBI is a better predictor of survival. Furthermore, ALBI showed more discriminative ability as indicated by c-index, both with UNOS (ALBI with UNOS: 0.753, CP with UNOS: 0.751), and without (ALBI: 0.623, CP: 0.616). However, this difference in discriminative power is small and the significance is unclear.

When broken into its components, albumin appears to contribute more to the predictive power of ALBI than bilirubin. Individually, albumin divided patients into 3 groups with statistically different median survivals (albumin 1: 46.0, albumin 2: 17.1, albumin 3: 9.1 months), while bilirubin did not (bilirubin 1: 15.6, bilirubin 2: 21.0, bilirubin 3: 5.8 months). Interestingly, median survivals in albumin groups were similar to median survivals in ALBI groups (ALBI 1: 46.7, ALBI 2: 19.2, ALBI 3: 8.8 months). Within BCLC, this trend continued. In BCLC A, B, and C, albumin split patients into groups with statistically different median survivals and these groups had similar survival to those of ALBI groups. Surprisingly, within BCLC B, median survival of albumin groups (within B, albumin 1: 46.7, albumin 2: 17.2, albumin 3: 13.5 months) had a greater degree of separation than ALBI (within B, ALBI 1: 38.5, ALBI 2: 22.7, ALBI 3: 12.6 months). This could be due to wide confidence intervals. Alternatively, this could be due to the lack of difference in median survival between bilirubin groups within BCLC B (within B, bilirubin 1: 17.7, bilirubin 2: 24.5, bilirubin 3: 25.4 months) with a log rank X^2^ of 0.1 (*p*-value = 0.90).

Cox proportional hazards analysis demonstrated the prognostic power of albumin alone as compared to bilirubin. Univariate analysis showed significantly greater hazard ratios in albumin (albumin 2:1 was 2.48, albumin 3:1 was 4.74) than bilirubin (bilirubin 2:1 was not significant, bilirubin 3:1 was 2.37). Albumin further showed more discriminative power as indicated by c-index (albumin: 0.640, bilirubin: 0.533). Albumin showed more discriminative ability than ALBI as indicated by c-index (albumin: 0.640, ALBI: 0.623), which held true with BCLC (with BCLC, albumin: 0.703, ALBI: 0.697). Again, this difference in discriminative power is small and the significance is unclear. Nevertheless, the unexpected nature of this finding warrants further investigation.

Our study has its strengths and limitations. Strengths include homogeneity of therapy, cohort size, and length of follow-up. Our study included a comprehensive and mature data set with prolonged follow-up that derives from patients receiving care at a large medical center by consensus decision of hepatologists, transplant and hepatobiliary surgeons, medical oncologists, and interventional radiologists. Furthermore, our study of 1000 patients expands on the analysis by Hickey et al. in that we have a larger population of Y90 patients, which gives us more statistical power in analyzing subgroups identified by ALBI or CP [4]. The proportion of ALBI 1 patients in our cohort was markedly increased as compared to Hickey et al. This enabled more confidence in conclusions drawn for patients with higher levels of hepatic reserve that are not eligible for resection. Limitations include the nature of the study as a single-center retrospective study. Furthermore, patient numbers continue to be a limitation, as only 44 patients were classified as CP C and 71 as ALBI 1. Conclusions must be drawn with care, due to possible statistical fluctuation. In assessing albumin and bilirubin, a further limitation is the use of CP cutoffs for albumin and bilirubin groups. These cutoffs created unequal bilirubin groups and the question arises whether a different cutoff point could add more power to survival predicted by bilirubin. Furthermore, while Johnson et al. created cutoffs at the 25th and 90th percentiles of ALBI grades in their study, these cutoffs do not hold true for our population, with cutoffs at approximately the 7th and 70th percentile. Inherently, the comparison of ALBI, BCLC, and Child-Pugh is difficult given that albumin is a central component of each of these staging systems and therefore they are all interrelated. Assessing the impact of albumin in each of these systems is difficult. UNOS was used in this context to assess tumor burden given that albumin is not a component of UNOS.

## 4. Materials and Methods

This study was compliant with the Health Insurance Portability and Accountability Act (HIPAA) and approved by the Northwestern University Institutional Review Board. A prospective database of consecutive patients with hepatocellular carcinoma (HCC) to be treated with Y90 was generated at Northwestern Memorial Hospital. Patients were considered for Y90 if they exhibited unresectable HCC as determined by a multidisciplinary team. Inclusion criteria included diagnosis of HCC by biopsy or imaging and treatment with Y90. These selection criteria identified a 1000-patient cohort to be used for this analysis.

### 4.1. Evaluation/Staging

Patients underwent pretreatment assessment including history, laboratory, and imaging work up. Baseline patient evaluation was performed using BCLC stage and CP class. Patients with no vascular invasion or extrahepatic metastases were classified as BCLC C only if specific HCC related symptoms were exhibited [12]. BCLC stages were calculated with Eastern Cooperative Oncology Group (ECOG) performance status. Patients were classified as cirrhotic only if the patient exhibited a nodular liver surface, splenomegaly, and/or thrombocytopenia. The ALBI score was calculated according to published criteria [11], which was then used to determine the ALBI grade.

ALBI Score = 0.66 × log10 ([bilirubin])—0.085 × [albumin]
ALBI Grade 1: ≤−2.60
ALBI Grade 2: −2.60 to −1.39
ALBI Grade 3: >−1.39

Albumin and bilirubin grades were assessed according to CP cutoffs.

Albumin Grade 1: >3.5 g/dLBilirubin Grade 1: <2 mg/dLAlbumin Grade 2: 2.8–3.5 g/dLBilirubin Grade 2: 2–3 mg/dLAlbumin Grade 3: <2.8 g/dLBilirubin Grade 3: >3 mg/dL

### 4.2. Transarterial Radioembolization

Transarterial yttrium-90 glass microsphere radioembolization (Y90) was performed according to a standardized published methodology [13,14]. This is beyond the scope of this manuscript.

### 4.3. Baseline Imaging

Patients were imaged at baseline using multiphasic magnetic resonance imaging (MRI) as per institutional standard, or multiphasic CT if MRI was not possible. HCC was diagnosed using American Association for the Study of Liver Diseases (AASLD) guidelines. Biopsy was performed if imaging features were not characteristic of HCC.

### 4.4. Statistical Analysis

Survival analysis was initiated on the date of Y90 and was right censored to curative therapy (resection or liver transplant). Kaplan Meier analysis was performed to assess overall and stratified survival. Differences in survival were evaluated using the log rank test.

Baseline patient characteristics were compared using the student’s t test or Mann-Whitney U test for continuous data, and X^2^ or Fisher exact test for categorical data. Proportional hazards assumption was validated visually with log–log plots and numerically with Schoenfeld weighted residuals. Cox model fit was evaluated using the likelihood ratio test (LR), Wald test, Harrell’s C (c-index), and corrected Akaike Information Criterion (AICc). The Wald test and LR test were used to assess within group homogeneity and monotonicity of gradients. The C-index was used to assess discriminatory ability. AICc was used to assess overall model quality and trade-off between generalizability and overfitting. ALBI and CP were compared using counts, survival analyses, and Cox proportional hazards analysis. Stratified survival analysis was performed for ALBI within CP, CP within ALBI, and for ALBI or CP within United Network for Organ Sharing (UNOS) HCC tumor grades to determine clinical relevance (ALBI within UNOS, CP within UNOS). UNOS was used in lieu of BCLC to limit collinearity as BCLC relies on CP. Cox proportional hazards, c-index, and corrected Akaike Information Criterion (AICc) were assessed to determine the relative impact of each factor on overall survival. Similar analyses were performed comparing albumin and bilirubin. A higher LR test, Wald test, and c-index score were associated with better fit; a lower AICc was associated with better fit. Analysis was performed using R Foundation for Statistical Computing, Vienna, Austria A *p*-value of <0.05 was considered significant.

## 5. Conclusions

Our data support the use of ALBI in our patient cohort as a measure of liver function as compared to CP. Previous studies suggest that ALBI is at least non-inferior to CP [4,5,6,7,8,9,10]. Numerous studies have shown that incorporation of ALBI into existing staging systems represents a valuable resource for evaluating treatment options for HCC patients [6,15,16,17]. Our study supports the use of ALBI in future analyses and trials as a tool to identify ALBI/CP discordant patients for whom therapy may be more beneficial or more harmful than indicated by CP. Our study supports the clinical use of ALBI in HCC as the marker of liver dysfunction. Additionally, our data shows that albumin appears to contribute more to the prognostic power of ALBI than bilirubin. Careful interpretation of this data is necessary given the interrelationship between albumin, ALBI, and CP. Nevertheless, there may be a role of using albumin alone to prognosticate HCC survival. Further investigation is needed into the individual roles of albumin and bilirubin on HCC prognosis.

## Figures and Tables

**Table 1:** Baseline Patient Characteristics.

Patient Factors		*n* = 1000 (%)
**Age (median, range)**	-	65 (20, 96)
**Gender**	M	752 (75.2)
	F	248 (24.8)
**Tumor focality**	Solitary	427 (42.7)
	Multifocal	573 (57.3)
**Tumor distribution**	Unilobar	639 (63.9)
	Bilobar	361 (36.1)
**Prior HCC Treatment**	None	889 (89)
	TACE	40 (4)
	RFA	15 (2)
	Resection	48 (5)
	Liver Transplant	6 (0.5)
**Tumor size (median, range)**	-	4.5 (2.7, 7.6)
**Portal vein thrombosis**	present	270 (27.0)
	absent	730 (73.0)
**Cirrhosis**	present	816 (81.6)
	absent	184 (18.4)
**Ascites**	present	242 (24.2)
	absent	758 (75.8)
**Extra-hepatic Metastases**	present	93 (9.3)
	absent	907 (90.7)
**Number Y90 treatments (median, range)**	-	1 (1, 8)
**Albumin (median, range)**	-	3 (2.6, 3.4)
**Bilirubin (median, range)**	-	1.1 (0.2, 93.0)
**Alkaline phosphatase (median, range)**	-	109 (16.0, 822.0)
**Alpha-fetoprotein (median, range)**	>100 ng/dL	409 (41)
	≤100 ng/dL	591 (59)
**Albumin Class**	1 (>3.5 g/dL)	188 (18.8)
	2 (2.8–3.5 g/dL)	490 (49.0)
	3 (<2.8 g/dL)	322 (32.2)
**Blirubin**	≤1 mg/dL	481 (48)
	>1mg/dL	519 (52)
**Bilirubin Class**	1	824 (82.4)
	2	124 (12.4)
	3	52 (5.2)
**ALBI**	1	71 (7.1)
	2	637 (63.7)
	3	292 (29.2)
**CP**	A	506 (50.6)
	B	450 (45.0)
	C	44 (4.4)
**BCLC**	A	263 (26.3)
	B	152 (15.2)
	C	541 (54.1)
	D	44 (4.4)
**ECOG**	0	557 (55.7)
	1	401 (40.1)
	2	42 (4.2)
**Combined UNOS**	T1/T2	368 (36.8)
	T3	169 (16.9)
	T4a	147 (14.7)
	T4b/N/M	316 (31.6)

**Table 2:** ALBI and CP Survival.

Subset	Stratification	Survival	Logrank *p*-Value
**CP A (*n* = 506)**	ALBI 1 (*n* = 435)	46.7 (43.8−not reached)	<0.001
	ALBI 2 (*n* = 69)	20.5 (17.3−24)	
	ALBI 3 (*n* = 2)	27.9 (not reached−not reached)	
**CP B (*n* = 450)**	ALBI 1 (*n* = 2)	1.9 (1.9−not reached)	<0.001
	ALBI 2 (*n* = 198)	14.7 (13.1−21)	
	ALBI 3 (*n* = 250)	9.1 (7.3−11)	
**CP C (*n* = 44)**	ALBI 1 (*n* = 0)	-	-
	ALBI 2 (*n* = 4)	not reached (4.6−not reached)	
	ALBI 3 (*n* = 40)	5.8 (4.4−not reached)	
**ALBI 1 (*n* = 71)**	CP-A (n = 69)	46.7 (43.8−not reached)	-
	CP-B (*n* = 2)	1.9 (1.9−not reached)	
	CP-C (*n* = 0)	-	
**ALBI 2 (*n* = 637)**	CP-A (*n* = 435)	20.5 (17.3−24)	0.006
	CP-B (*n* = 198)	14.7 (13.1−21)	
	CP-C (*n* = 4)	not reached (4.6−not reached)	
**ALBI 3 (*n* = 292)**	CP-A (*n* = 2)	27.9 (not reached−not reached)	0.314
	CP-B (*n* = 250)	9.1 (7.3−11)	
	CP-C (*n* = 40)	5.8 (4.4−not reached)	

**Table 3:** ALBI and CP Survival within UNOS stages.

Subset	Stratification	Survival	Logrank *p*-Value
**T1/T2 (*n* = 368)**	ALBI 1 (*n* = 40)	Not reached	<0.001
	ALBI 2 (*n* = 225)	39.5 (32.8−80.2)	
	ALBI 3 (*n* = 103)	20.3 (14.3−29.1)	
	CP A (*n* = 194)	61.0 (39.5−not reached)	<0.001
	CP B (*n* = 152)	27.1 (20.3−31.1)	
	CP C (*n* = 22)	Not reached	
**T3 (*n* = 169)**	ALBI 1 (*n* = 13)	43.8 (14.0−not reached)	<0.001
	ALBI 2 (*n* = 121)	35.0 (24.9−41.6)	
	ALBI 3 (*n* = 35)	11.2 (6.8−not reached)	
	CP A (*n* = 102)	25.7 (25.5−44.4)	0.011
	CP B (*n* = 63)	19.9 (14.7−38.3)	
	CP C (*n* = 2)	14.8 (not reached−not reached)	
**T4a (*n* = 147)**	ALBI 1 (*n* = 7)	38.5 (30.3−not reached)	<0.001
	ALBI 2 (*n* = 100)	14.6 (11.3−21.0)	
	ALBI 3 (*n* = 40)	9.3 (6.1−13.5)	
	CP A (*n* = 75)	17.1 (13.3−22.7)	<0.001
	CP B (*n* =65)	11.5 (6.8−15.1)	
	CP C (*n* = 7)	3.6 (1.6−not reached)	
**T4b/N/M (*n* = 316)**	ALBI 1 (*n* = 11)	14.2 (6.9−not reached)	<0.001
	ALBI 2 (*n* = 191)	8.7 (7.7−11.3)	
	ALBI 3 (*n* = 114)	4.8 (3.4−6.4)	
	CP A (*n* = 135)	10.4 (8.7−13.3)	<0.001
	CP B (*n* = 170)	5.6 (4.6−6.7)	
	CP C (*n* = 11)	2.5 (2.3−not reached)	

**Table 4:** Cox Proportional Hazards.

		HR (95% CI)	p value	c-index	AICc	LR test	Wald test
**ALBI**	1			0.623	6155.8	102.6	98.1
	2	3.39 (1.75, 6.57)	<0.001				
	3	7.58 (3.89, 14.79)	<0.001				
**CP**	1			0.616	6184.7	73.8	74.4
	2	2.04 (1.71, 2.43)	<0.001				
	3	3.27 (2.08, 5.14)	<0.001				
**ALBI** **with UNOS**	1			0.753	5926.7	337.8	313.3
	2	2.80 (1.44, 5.43)	0.002				
	3	5.70 (2.92, 11.14)	<0.001				
**CP** **with UNOS**	1			0.751	5936.3	328.2	305.9
	2	1.84 (1.54, 2.19)	<0.001				
	3	4.57 (2.90, 7.22)	<0.001				
**Albumin**	1			0.640	6136.6	121.8	108.2
	2	2.48 (1.81, 3.41)	<0.001				
	3	4.74 (3.44, 6.54)	<0.001				
**Bilirubin**	1			0.533	6240.9	17.5	22.1
	2	1.09 (0.82, 1.44)	0.546				
	3	2.37 (1.66, 3.40)	<0.001				
**Albumin** **with BCLC**	1			0.703	6045.6	218.9	184.0
	2	2.31 (1.69, 3.17)	<0.001				
	3	4.03 (2.91, 5.58)	<0.001				
**Bilirubin** **with BCLC**	1			0.656	6121.5	142.9	124.1
	2	1.10 (0.83, 1.45)	0.504				
	3	2.62 (1.68, 4.10)	<0.001				
**ALBI** **with BCLC**	1			0.697	6061.1	203.4	177.9
	2	2.89 (1.58, 2.95)	0.002				
	3	5.93 (3.03, 11.61)	<0.001				

**Table 5:** Albumin and Bilirubin Survival within BCLC stages.

Subset	Stratification	Survival	Logrank *p*-Value
**BCLC A (*n* = 263)**	Albumin 1 (*n* = 68)	not reached	<0.001
	Albumin 2 (*n* = 134)	35.9 (30.2−61.0)	
	Albumin 3 (*n* = 61)	25.1 (17.5−31.1)	
	Bilirubin 1 (*n* = 215)	35.9 (32.1−61.0)	0.07
	Bilirubin 2 (*n* = 40)	27.8 (17.1−not reached)	
	Bilirubin 3 (*n* = 8)	12.9 (7.8−not reached)	
	ALBI 1 (*n* = 31)	not reached	<0.001
	ALBI 2 (*n* = 174)	37.3 (33.5−80.2)	
	ALBI 3 (*n* = 58)	21.0 (17.5−34.5)	
**BCLC B (*n* = 152)**	Albumin 1 (*n* = 30)	46.7 (30.3−not reached)	<0.001
	Albumin 2 (*n* = 83)	17.2 (12.0−24.9)	
	Albumin 3 (*n* = 39)	13.5 (6.3−21.4)	
	Bilirubin 1 (*n* = 133)	17.7 (15.1−25.5)	0.90
	Bilirubin 2 (*n* = 15)	24.5 (15.1−not reached)	
	Bilirubin 3 (*n* = 4)	25.4 (16.1−not reached)	
	ALBI 1 (*n* = 7)	38.5 (30.3−not reached)	0.001
	ALBI 2 (*n* = 114)	22.7 (17.1−29.9)	
	ALBI 3 (*n* = 31)	12.6 (6.3−16.3	
**BCLC C (*n* = 541)**	Albumin 1 (*n* = 90)	20.1 (15.2−not reached)	<0.001
	Albumin 2 (*n* = 268)	13.3 (11.3−14.7)	
	Albumin 3 (*n* = 183)	7.3 (6.4−8.8)	
	Bilirubin 1 (*n* = 469)	11.5 (10.4−13.3)	<0.001
	Bilirubin 2 (*n* = 60)	8.0 (6.7−21.0)	
	Bilirubin 3 (*n* = 12)	2.7 (1.1−not reached)	
	ALBI1 (*n* = 33)	not reached	<0.001
	ALBI2 (*n* = 345)	13.8 (12.0−14.9)	
	ALBI3 (*n* = 163)	6.7 (5.7−8.8)	
**BCLC D (*n* = 44)**	Albumin 1 (*n* = 0)	−	−
	Albumin 2 (*n* = 5)	5.0 (4.6−not reached)	
	Albumin 3 (*n* = 39)	10.3 (3.6−not reached)	
	Bilirubin 1 (*n* = 7)	not reached	0.06
	Bilirubin 2 (*n* = 9)	16.0 (4.8−not reached)	
	Bilirubin 3 (*n* = 28)	4.6 (2.5−not reached)	
	ALBI 1 (*n* = 0)	−	−
	ALBI 2 (*n* = 4)	not reached	
	ALBI 3 (*n* = 40)	5.8 (4.4−not reached)	

**Table 6:** Cox Proportional hazards of albumin and bilirubin as continuous variables.

Group		Hazard Ratio	*p-*Value
T1/T2	Albumin	0.26 (0.17, 0.39)	<0.001
	Bilirubin	1.27 (1.09, 1.47)	0.002
T3	Albumin	0.21 (0.12, 0.35)	<0.001
	Bilirubin	0.98 (0.93, 1.04)	0.55
T4a	Albumin	0.46 (0.33, 0.64)	<0.001
	Bilirubin	1.42 (1.14, 1.76)	0.002
T4b/N/M	Albumin	0.47 (0.37, 0.6)	<0.001
	Bilirubin	1.61 (1.41, 1.85)	<0.001
All groups (T1-M)	Albumin	0.34 (0.29, 0.4)	<0.001
	Bilirubin	1 (0.98, 1.02)	0.921

## Supplementary Materials

The following are available online at https://www.mdpi.com/2072-6694/11/6/879/s1, Figure S1: ALBI and CP Survival Analysis, Figure S2: ALBI and CP Survival within UNOS Stages, Figure S3: UNOS Combined Groups.
