# Supplementary material for: Prognostic Role of Albumin, Bilirubin, and ALBI Scores: Analysis of 1000 Patients with Hepatocellular Carcinoma Undergoing Radioembolization
Source: Cancers (Basel). 2019 Jun 24;11(6):879. doi: 10.3390/cancers11060879 (PMC6627853; doi:10.3390/cancers11060879)
Supplement: Supplementary file 1 [file cancers-11-00879-s001.pdf]

Supplementary materials

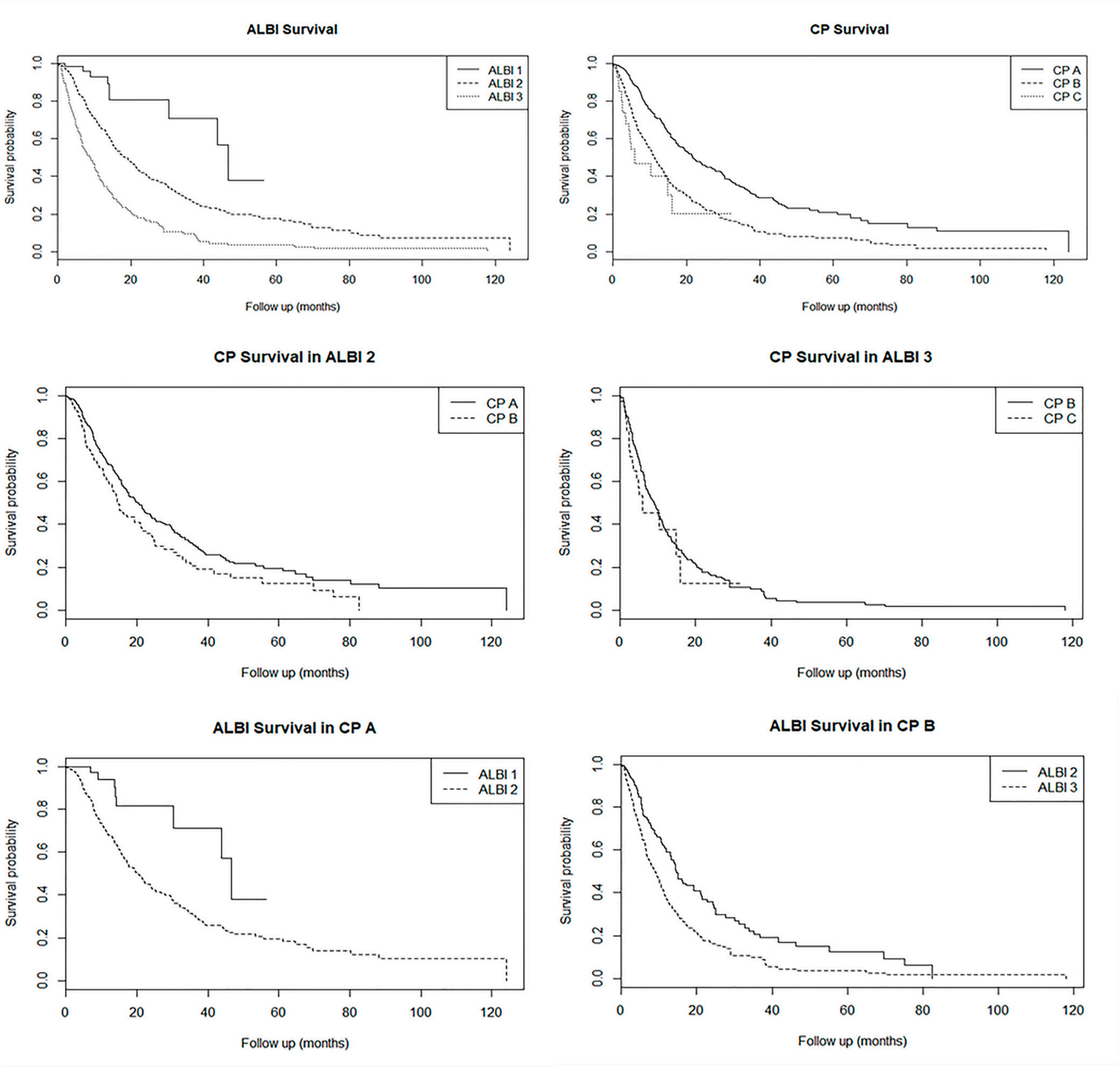

Figure S1. ALBI, CP Survival Analysis.

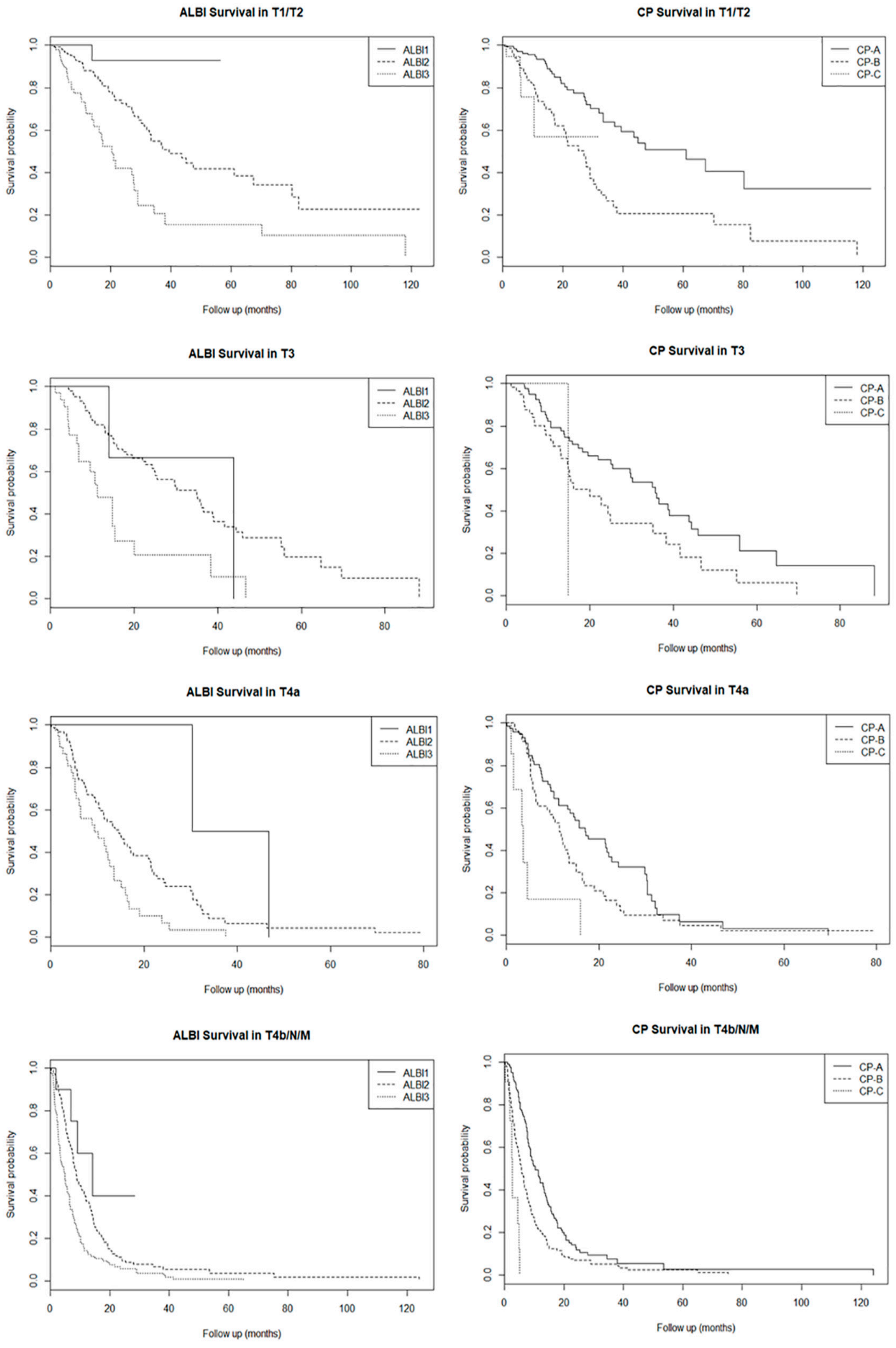

Figure S2. ALBI, CP Survival in UNOS.

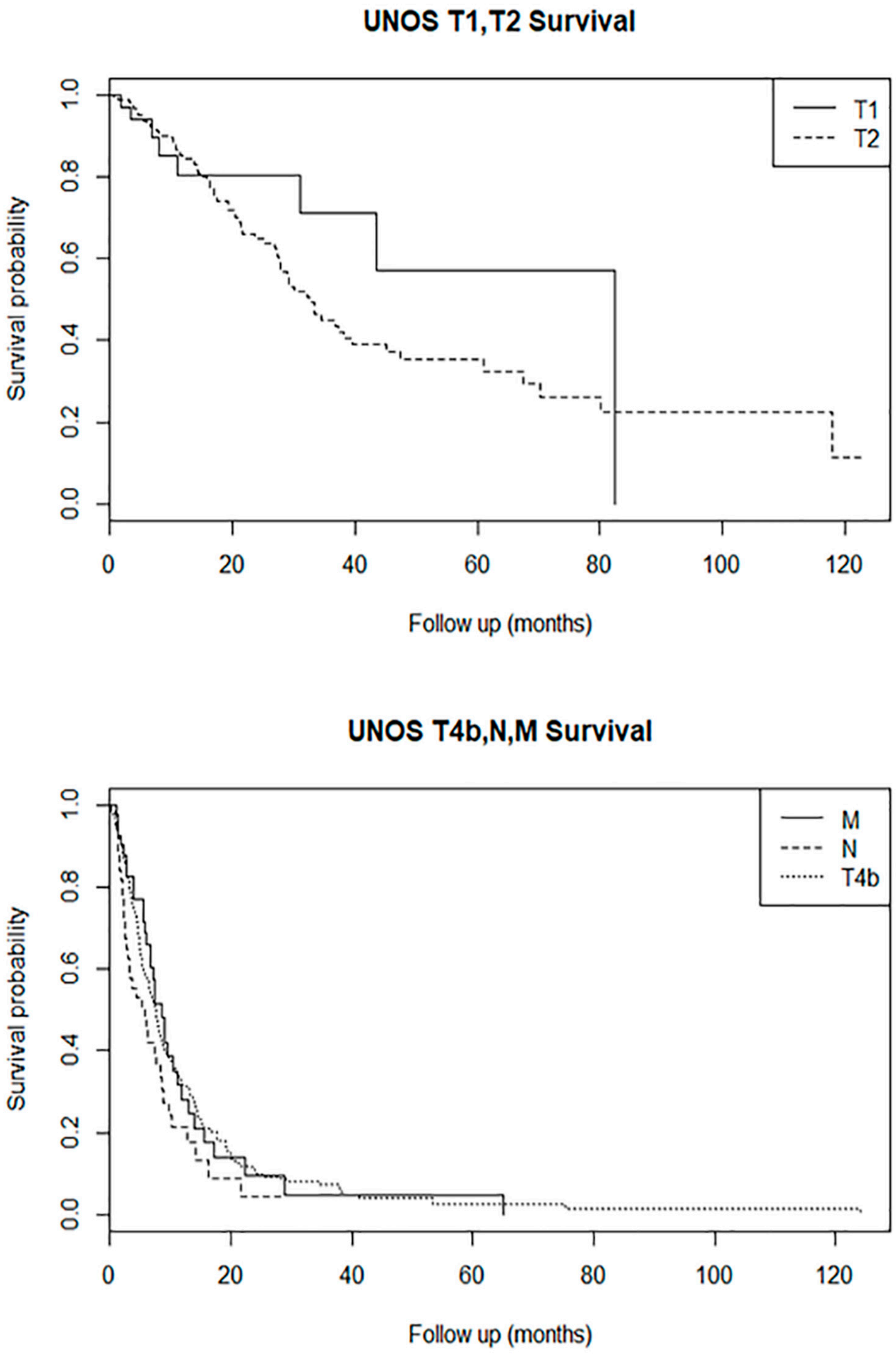

Figure S3. UNOS T1/T2 and T4b/N/M.
